# Comment on “Management of Atrial Fibrillation in Critically Ill Patients”

**DOI:** 10.1155/2016/8985161

**Published:** 2016-08-24

**Authors:** Roger Jelliffe

**Affiliations:** Children's Hospital of Los Angeles, University of Southern California School of Medicine, 4640 Hollywood Boulevard, Los Angeles, CA 90027, USA

I wish to report a significant misstatement in an article titled “Management of Atrial Fibrillation in Critically Ill Patients” [1].

On the left column at the bottom of page 5, there is a section discussing the use of digoxin. The authors say “despite its efficacy in controlling resting heart rates, it is not a converter” and make no further statement. They then go on toward the upper part of the following right hand column to say “serum digoxin levels (measured at least 6 hours after the last dose) may be helpful to corroborate the diagnosis of toxicity but are not recommended for routine use”, and they refer to an article of mine (their reference [73]) in apparent support of this. The reference is R. W. Jelliffe, “Some Comments and Suggestions Concerning Population Pharmacokinetic Modeling, Especially of Digoxin, and Its Relation to Clinical Therapy”, Therapeutic Drug Monitoring, vol 34, pp. 368–377, 2012.

*That Is a Misstatement*. In the first place, I would suggest that managing atrial fibrillation (AF) in critically ill patients is not at all routine use, and I strongly advocate the use of serum levels to monitor and guide digoxin therapy there.

The authors, however, appear to be unaware that, in the very article they cite, I also strongly advocate the use of D-optimal design strategies to obtain serum levels so that one can obtain maximally informative data about the behavior of the drug. For example, in the article they refer to, their reference [73], I specifically suggest getting digoxin levels about 5 min after a 15 min intravenous infusion or about 1.5 to 1.75 hours after an oral dose, along with a trough.

In that* same* article I also specifically discuss the use of digoxin to convert patients with AF to sinus rhythm, with a brief review of the available literature on the subject (none of which is mentioned in their review), and I also discuss some patients I have managed and converted to sinus rhythm, at least for a period of several weeks, which might serve as a starting point in a future further investigation.

It is distressing to be so misquoted about specific strategies for getting serum levels. I invite the authors and the readers to read the paper they refer to and to think further, and for themselves, about the management of AF in critically ill patients.

The authors might have mentioned in their review that I successfully converted three of the four patients I describe in my article, one with well-established chronic atrial flutter, with digitoxin or digoxin, using specific pharmacokinetic software guidance designed for the task. The response of the patients, including the one who was not converted, correlated very well not with serum digoxin concentrations but with the computed concentrations in the peripheral nonserum compartment, as described in that article.

One other point to mention is that the use of capable software is essential to make sense out of the serum concentration data and to compute the concentration of digoxin in the patient's peripheral nonserum compartment. The Bestdose (formerly Rightdose) software [2, 3] was developed with just this sort of application in mind. To my knowledge, it is the only software capable of doing this and of developing dosage regimens to hit individualized therapeutic target goals specifically with maximum precision, using nonparametric pharmacokinetic models [4] and multiple model dosage design [5].
